# The N. Y. World’s Fair

**Published:** 1889-10

**Authors:** 


### THE N. Y. WORLD'S FAIR.

The almost unprecedentedly cold and dreary weather which followed hard upon the terrible southeaster, which made wreck of so many sea-side resorts and summer homes along the coasts of New York and New Jersey, has caused thousands of our citizens to beat a hasty retreat to their city habitations, and New York is all alive again.

The all-absorbing topic of a general interest is the contemplated World's Fair of some two years' hence. The committee having in charge the selection of a site, whereon to erect the necessary buildings and display the various features of the world-centered exhibition, so full of promise, after much discussion have agreed upon Morningside and Riverside Parks. The estimated cost of the requisite buildings is $6,300,000, the whole toinclude a space of 62 acres of land, which it is practicable to furnish at the suggested localities.

The opposition to the use of any part of Central Park has led to its abandonment.

It is anticipated that the 1892 Fair will be the finest display of the kind ever held anywhere ; and New York, which never does anything by halves, is determined to make it so.

